# The complete chloroplast genome sequence of *Berchemia racemosa* Siebold & Zucc. (Rhamnaceae), a rare plant species in Korea

**DOI:** 10.1080/23802359.2022.2161329

**Published:** 2023-01-02

**Authors:** Joon Moh Park, Jachoon Koo

**Affiliations:** a Forest Resource Research Division, Jeollabuk-do Forest Environment Research Institute, Jinan, South Korea; b Division of Science Education and Institute of Fusion Science, College of Education, Jeonbuk National University, Jeonju, South Korea

**Keywords:** *Berchemia racemosa*, complete chloroplast genome, phylogeny, Rhamnaceae

## Abstract

*Berchemia racemosa* Siebold & Zucc., 1845 is a rare species distributed in restricted areas in the western Korean peninsula. In this study, the complete chloroplast genome (plastome) of *B. racemosa* was sequenced and assembled by Illumina paired-end sequencing. The plastome of *B. racemosa* was 161,187 bp in length and was quadripartite in structure, including a large single-copy (LSC) region of 89,503 bp, a small single-copy (SSC) region of 18,214 bp, and two inverted repeats of 26,735 bp. The GC content was 37.2%. The plastome of *B. racemosa* contains 130 genes, including eight ribosomal RNA (rRNA) genes, 37 transfer RNA (tRNA) genes, and 85 protein-coding genes. Phylogenetic analysis using complete genome sequences showed that *B. racemosa* is most closely related to *Berchemia flavescens*.

## Introduction

*Berchemia racemosa* Siebold & Zucc., 1845 is a deciduous vine in the Rhamnaceae family that is native to Korea, Japan, and Taiwan. In Korea, *B. racemosa* is a rare plant distributed only in a limited region in Jeollabuk-do Province. Its natural habitat in Jeollabuk-do Province is designated as a nature reserve. Here, we analyzed the complete chloroplast genome (plastome) sequence of *B. racemosa* to provide valuable information for genetic diversity and phylogenetic relationships between *B. racemosa* and other species in the Rhamnaceae family.

## Materials and methods

### Sampling and genome sequencing

**Figure 1.**
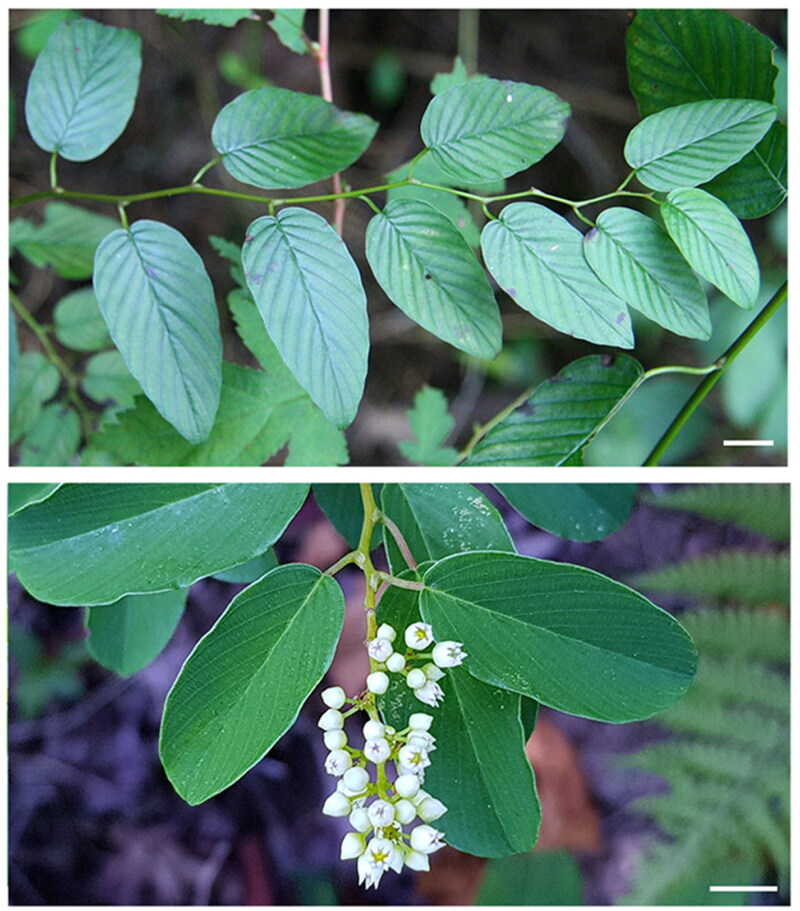
Photographs of *B. racemose* plants showing a typical branching (upper panel) and floral structure (lower panel). Images were taken using a digital camera at Wolmyeong Park (Gunsan, Jeollabuk-do Province, South Korea). Scale bars represent 1 cm.

Plant materials of *B. racemosa* were sampled from Gunsan, Jeollabuk-do Province, Korea (126°41′23.10″E, 35°58′41.90″N). Each specimen was imaged using a digital camera to record the information about the sampling sites in natural habitat (Figure 1). The specimens of *B. racemosa* were identified by Joon Moh Park and deposited in the Jeollabuk-do Forest Environment Research Institute, South Korea (https://forest.jb.go.kr/; contact person, Joon Moh Park; E-mail, joonmoh@korea.kr) under the voucher number JFERI0020-2. Research plan for sample collection and experimental methods in this study has passed the ethical survey of the Plant Ethics Committee from Jeollabuk-do Forest Environment Research Institute. Total genomic DNA was extracted from the fresh leaf of *B. racemosa* using an Exgene Plant SV kit (GeneAll Inc., Seoul, South Korea). The DNA was mechanically fragmented to create the sequencing library, and both ends of the DNA were then ligated with adaptors using the TruSeq DNA PCR Free kit (Macrogen Inc., Seoul, South Korea). A paired-end library with an insert size between 350 and 450 bp (Supplementary Tables 1–3) was sequenced using the Illumina HiSeq X platform (Macrogen Inc., Seoul, South Korea).

### Assembly and annotation of chloroplast genome

The complete plastome was assembled using NOVOPlasty v.4.3.1 (Dierckxsens et al. 2017). Gene annotation was performed using GeSeq v.1.59 (Tillich et al. 2017) with options of Chloe v. 0.1.0., BLATN, and BLATX. BLAST was used to further identify positions of inverted repeat (IR) regions by searching against the published plastome database. A circular map of the complete plastome was generated by CPGView software (http://www.1kmpg.cn/cpgview/). The genomic DNA of *B. racemosa* was deposited in the Jeollabuk-do Forest Environment Research Institute (contact person, Joon Moh Park; E-mail, joonmoh@korea.kr) under the voucher number JFERI-DNA0020-2. All biological samples and research in this study had been approved by the Ethics Committee of Jeollabuk-do Forest Environment Research Institute. To clarify the phylogenetic position of *B. racemosa*, the complete plastome sequences of 47 Rosales species and two outgroups (*Castanea mollissima* and *Cucurbita moschata*) were downloaded from GenBank and aligned using MAFFT v7.3 (Katoh and Standley 2013). Spurious matches or poorly aligned regions were removed from the multiple sequence alignment by TrimmAl v.1.2 (Capella-Gutiérrez et al. 2009). The resulting aligned sequences of 127,121 bp were analyzed by the maximum-likelihood (ML) method using IQ-Tree (Nguyen et al. 2015) with a TVM + F+R4 substitution model as a best-fit model, 1000 replicates of ultrafast bootstrap, and SH-aLRT branch support.

## Results

### Chloroplast genome features

**Figure 2.**
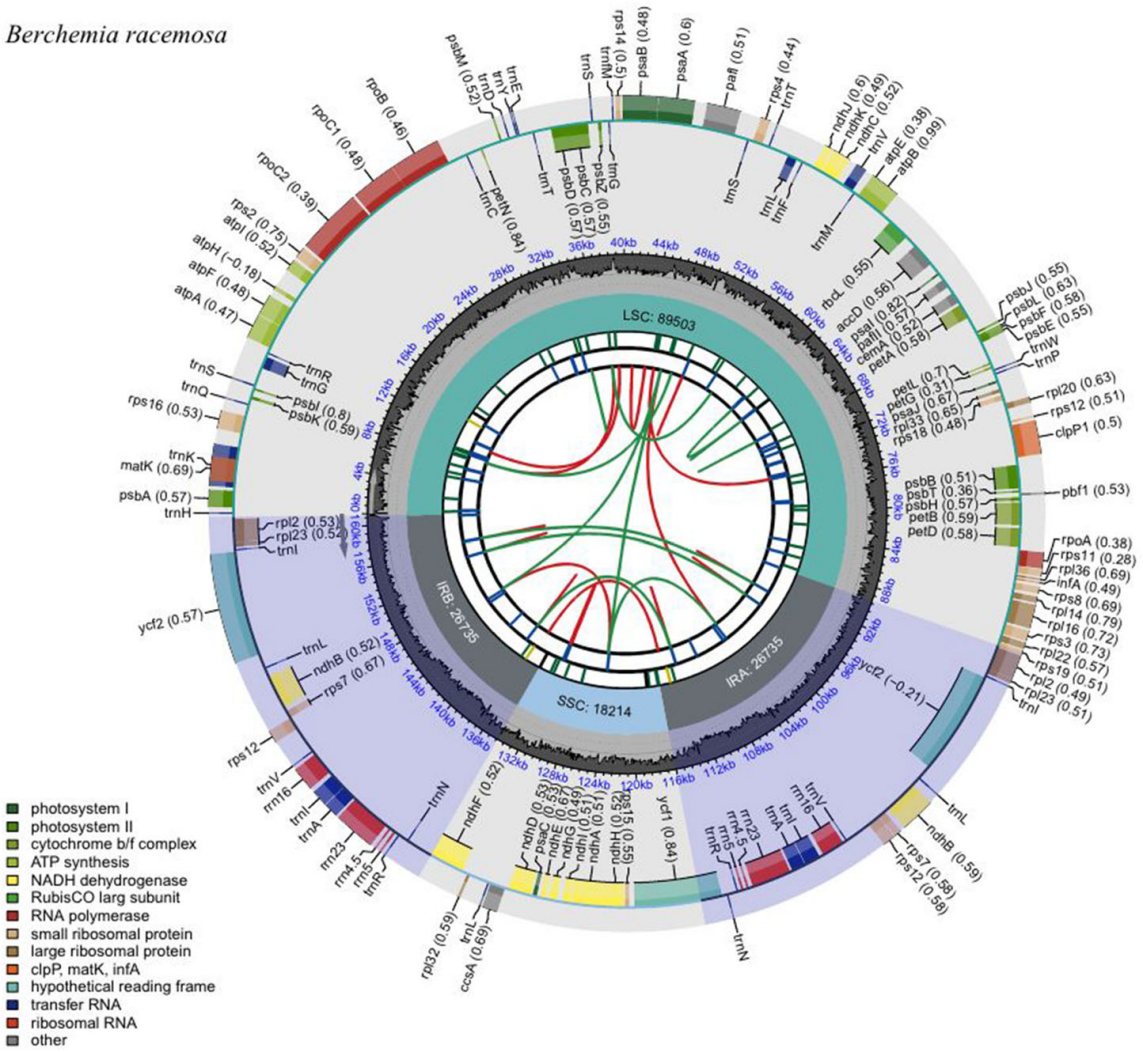
Schematic map of *B. racemosa* complete chloroplast genome constructed by CPGview (http://www.1kmpg.cn/cpgview/). The map contains six tracks. From the center to outward, the first track shows the dispersed repeats. The second track shows the long tandem repeats as short blue bars. The third track shows the short tandem repeats or microsatellite sequences as short bars with different colors. The small single-copy (SSC), inverted repeat (IRa and IRb), and large single-copy (LSC) regions are shown on the fourth track. The GC content along the genome is plotted on the fifth track. The genes are shown on the sixth track. The optional codon usage bias is displayed in the parenthesis after the gene name. The transcription directions for the inner and outer genes are clockwise and anticlockwise, respectively. The functional classification of the genes is shown in the bottom left corner.

**Figure 3.**
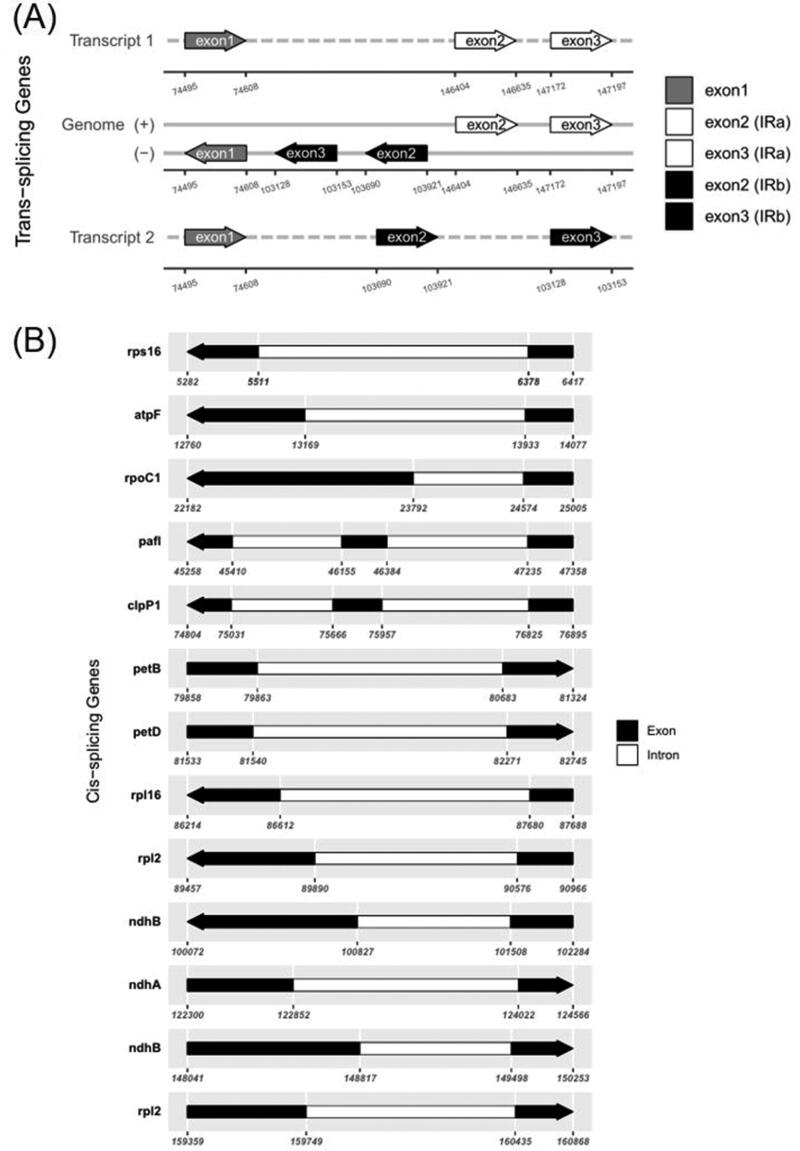
Schematic map of the trans-splicing gene *rps12* (A) and cis-splicing genes (B) in the chloroplast genome of *B. racemosa*. The exons are shown in black; the introns are shown in white. The arrow indicates the sense direction of the gene. The map was generated using CPGview software (http://www.1kmpg.cn/cpgview/).

The complete plastome of *B. racemosa* (GenBank accession number ON749761) is 161,187 bp with 37.2% GC content. It is composed of a pair of IR regions of 26,735 bp, a large single-copy (LSC) region of 89,503 bp, and a small single-copy (SSC) region of 18,214 bp (Figure 2). The gene structure of the plastome was nearly identical to that of other Rhamnaceae species (Ma et al. 2017). A total of 130 genes were annotated in the plastome, comprising eight ribosomal RNA (rRNA) genes, 37 transfer RNA (tRNA) genes, and 85 protein-coding genes (PCGs). The *rps12* is a trans-spliced gene. The 5′ exon of this gene is found in the LSC region, while the 3′ exon is duplicated in the IR regions (Figure 3(A)). Thirteen genes, including *rps16*, *atpF*, *rpoC1*, *pafI*, *clpP1*, *petB*, *petD*, *rps16*, *rpl2*, *ndhB*, *ndhA*, *ndhB*, and *rpl2*, contain one or two introns (Figure 3(B)).

Whole plastome sequence of seven Rhamnaceae species was aligned to compare the variations of gene structure within the family. The plastome of these species showed high similarity in terms of the number, length, and arrangement of genes. In particular, the plastome sequence of *B. racemosa* showed the highest sequence similarity of 98.7% with *Berchemia flavescens* (Zhu et al. 2019), except for the inverted orientation of a 13,092-bp fragment from 115,721 to 128,812 bp.

### Phylogenetic analysis

**Figure 4.**
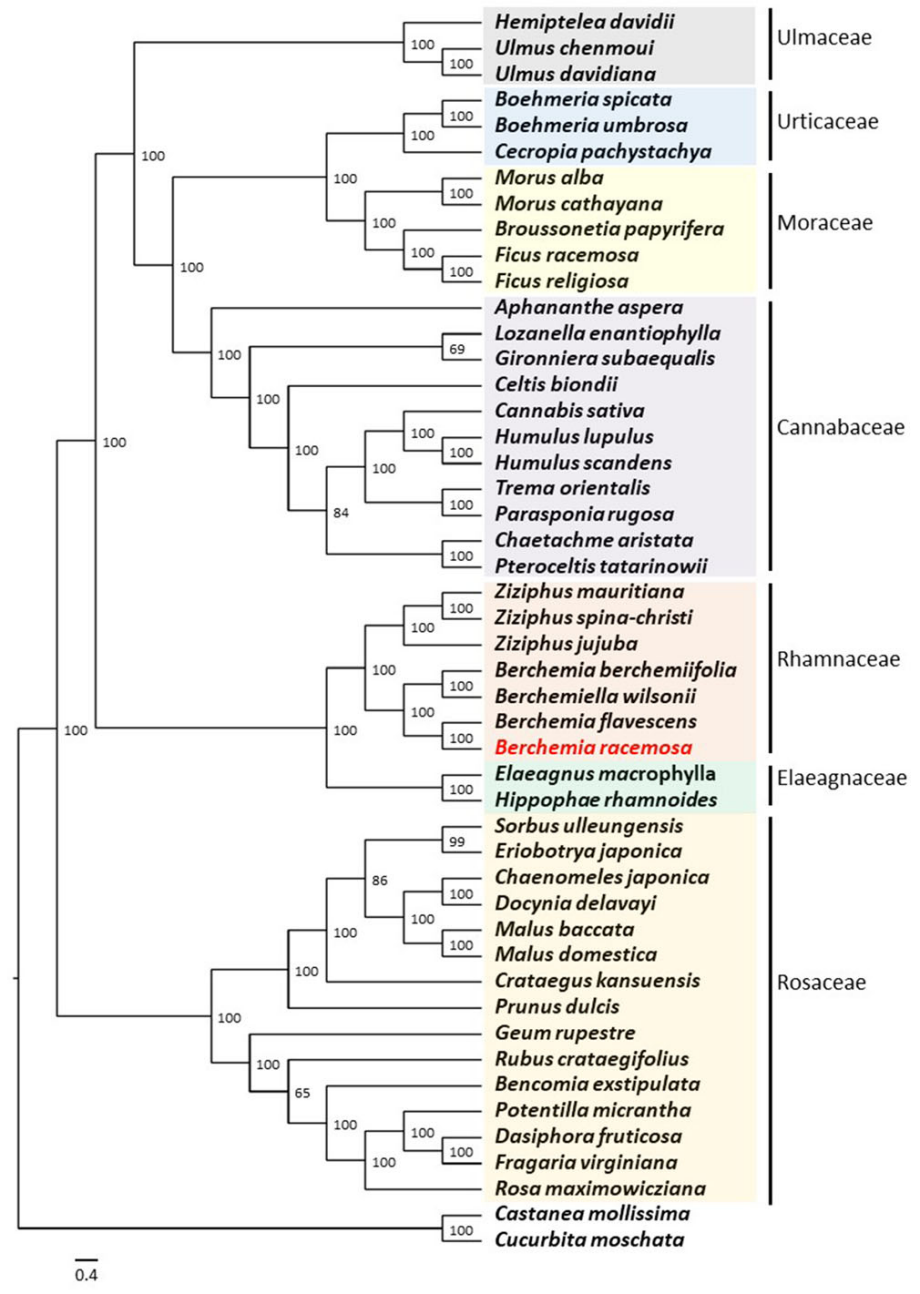
Maximum-likelihood (ML) tree showing the relationship among *B. racemosa* and representative species within Order Rosales based on the complete chloroplast genome sequences. The following sequences were used: *Aphananthe aspera* (NC_039726; Zhang et al. 2018), *Bencomia exstipulata* (NC_039924), *Berchemia berchemiifolia* (NC_037477; Cheon et al. 2018), *Berchemia flavescens* (MK460212; Zhu et al. 2019), *Berchemia racemosa* (ON749761; this study), *Berchemiella wilsonii* (KY926621; Wang et al. 2018), *Boehmeria spicata* (NC_036989; Huang et al. 2019), *Boehmeria umbrosa* (NC_036990; Huang et al. 2019), *Broussonetia papyrifera* (KX828844), *Cannabis sativa* (KR363961; Oh et al. 2016), *Cecropia pachystachya* (NC_039763; Wu et al. 2017), *Celtis biondii* (NC_039727; Zhang et al. 2018), *Chaenomeles japonica* (KT932966), *Chaetachme aristata* (MH118120; Zhang et al. 2018), *Crataegus kansuensis* (MF784433; Zhang et al. 2020), *Dasiphora fruticosa* (MF683841; Zhao et al. 2018), *Docynia delavayi* (KX499860; Zhang et al. 2017), *Elaeagnus macrophylla* (KP211788; Choi et al. 2015), *Eriobotrya japonica* (KT633951; Shen et al. 2016), *Fragaria virginiana* (KY085911), *Ficus racemosa* (KT368151), *Ficus religiosa* (KY416513; Bruun-Lund et al. 2017), *Geum rupestre* (NC_037392; Duan et al. 2018), *Hemiptelea davidii* (MK070168; Liu et al. 2019), *Hippophae rhamnoides* (NC_035548; Chen and Zhang 2017), *Humulus lupulus* (KT266264; Vergara et al. 2016), *Humulus scandens* (NC_039730; Zhang et al. 2018), *Gironniera subaequalis* (MH118121; Zhang et al. 2018), *Lozanella enantiophylla* (MH118123; Zhang et al. 2018), *Malus baccata* (KX499859; Zhang et al. 2017), *Malus domestica* (KY818915), *Morus alba* (KU981119), *Morus cathayana* (MW465956), *Parasponia rugosa* (NC_039732; Zhang et al. 2018), *Potentilla micrantha* (HG931056; Ferrarini et al. 2013), *Prunus dulcis* (NC_034696), *Pteroceltis tatarinowii* (NC_039733; Zhang et al. 2018), *Rosa maximowicziana* (MG727865; Jeon and Kim 2019), *Rubus crataegifolius* (NC_039704; Yang et al. 2017), *Sorbus ulleungensis* (NC_037022), *Trema orientalis* (NC_039734; Zhang et al. 2018), *Ulmus davidiana* (NC_032718), *Ulmus chenmoui* (MG581403; Zhang et al. 2019), *Ziziphus jujuba* (NC_030299; Ma et al. 2017), *Ziziphus mauritiana* (NC_037151), and *Ziziphus spina-christi* (KY628305). *Castanea mollissima* (KY951992) and *Cucurbita moschata* (NC_036506) were included as outgroups. The numbers on the nodes indicate bootstrap values from 1000 replicates. The scale bar represents the number of substitutions per site.

Complete plastome sequences of 47 species from seven families in Order Rosales were utilized to explore the phylogenetic position of *B. racemosa*. The reconstructed phylogenetic tree showed that *B. racemosa* is most closely related to *Berchemia flavescens* (Zhu et al. 2019) with high support (BS = 100), both of which belong to the genus *Berchemia* in the Rhamnaceae family (Figure 4). It also indicated that Rhamnaceae are monophyletic and sister to the Elaeagnaceae family. In addition, our phylogenetic tree confirmed that all seven families formed monophyletic groups.

## Discussion and conclusions

*B. racemosa* is a rare plant distributed only in a limited region in Jeollabuk-do Province, South Korea. In this study, we reported the complete plastome of *B. racemosa* together with its genome features. Phylogenetic analysis based on the complete plastome strongly supported earlier study that Rhamnaceae are monophyletic and sister to the Elaeagnaceae family (Cheon et al. 2018). Also, *B. racemosa* is particularly closely related to *Berchemia flavescens* (BS = 100). This study provides valuable insights into the phylogenetic and evolutionary position of *B. racemosa* in the Rhamnaceae family and Order Rosales.

## Supplementary Material

Supplemental Material

## Data Availability

The genome sequence data that support the findings of this study are openly available in NCBI (https://www.ncbi.nlm.nih.gov) under the accession no. ON749761. The associated BioProject, SRA, and Bio-Sample numbers are PRJNA835661, SRR21615884, and SAMN30910605, respectively.
