# Supplementary material for: The complete chloroplast genome sequence of Berchemia racemosa Siebold & Zucc. (Rhamnaceae), a rare plant species in Korea
Source: Mitochondrial DNA B Resour. 2023 Jan 2;8(1):69–72. doi: 10.1080/23802359.2022.2161329 (PMC9817123; doi:10.1080/23802359.2022.2161329)
Supplement: Supplemental Material [file TMDN_A_2161329_SM4007.docx]

**PLASTOME ANNOUNCEMENT**

**The complete chloroplast genome sequence of *Berchemia racemosa* Siebold & Zucc. (Rhamnaceae), a rare plant species in Korea**

Joon Moh Park^1^ and Jachoon Koo^2^

^1^Forest Resource Research Division, Jeollabuk-do Forest Environment Research Institute, Jinan 55454, South Korea; joonmoh@korea.kr

^2^Division of Science Education and Institute of Fusion Science, College of Education, Jeonbuk National University, Jeonju 54896, South Korea; [jkoo@jbnu.ac.kr](mailto:jkoo@jbnu.ac.kr)

Correspondence:

Jachoon Koo

Tel: +82-63-270-2784

Fax: +82-63-270-2781

E-mail: jkoo@jbnu.ac.kr

**Supplementary Table 1. Raw data statistics.**


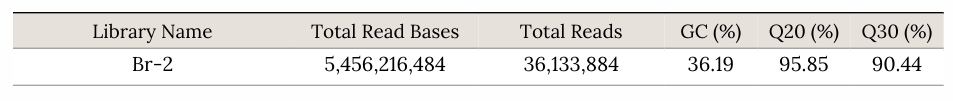


***** The total number of bases, reads, GC (%), Q20 (%), Q30 (%) are calculated for *B. racemosa* library (Br-2) sample.

**Supplementary Table 2. Filtered data statistics.**


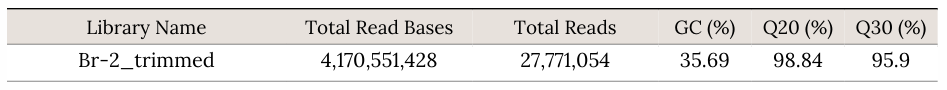


* Trimmomatic was used to remove adapter sequences and low quality reads in order to reduce biases in analysis. The total number of bases, reads, GC (%), Q20 (%), and Q30 (%) were calculated for the Br-2 sample after filtering.

**Supplementary Table 3. Overall mapping statistics.**


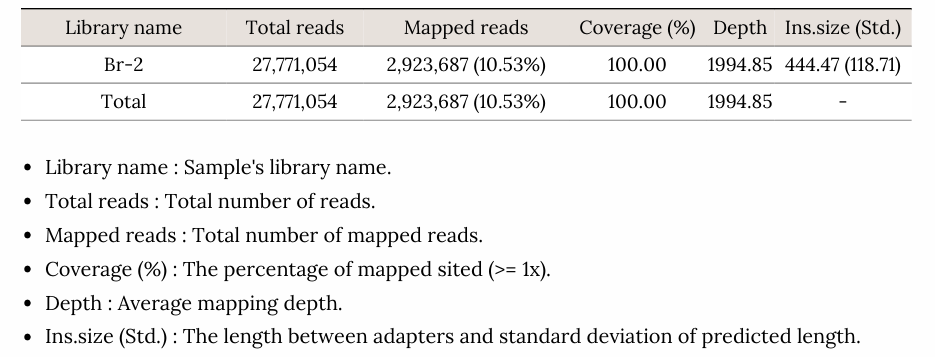


* In order to identify the insert size of raw data and how much reads were used in assembly, raw data reads were mapped to an assembly result. After mapping, the necessary statistics were calculated.
